# Variation in Current Guidelines for Fluoride Varnish Application for Young Children in Medical Settings in the United States

**DOI:** 10.3389/fpubh.2022.785296

**Published:** 2022-03-04

**Authors:** Sarah L. Goff, Grace Gahlon, Kimberley H. Geissler, Andrew W. Dick, Ashley M. Kranz

**Affiliations:** ^1^University of Massachusetts Amherst School of Public Health and Health Sciences, Amherst, MA, United States; ^2^RAND Corporation, Arlington, VA, United States; ^3^RAND Corporation, Boston, MA, United States

**Keywords:** oral health, children, fluoride varnish, guidelines & recommendations, variation

## Abstract

**Background:**

The United States Preventive Services Task Force recommends that medical providers apply fluoride varnish (FV) to the teeth of all children under 6 years of age, but fewer than 10% of eligible children receive FV as recommended. Prior studies suggest that variation in clinical guidelines is associated with low uptake of other evidence-based health-related interventions, but consistency of national guidelines for the delivery of FV in medical settings is unknown.

**Methods:**

Eligible guidelines for application of FV in medical settings for children under 6 years of age were published in the past 10 years by national pediatric or dental professional organizations or by national public health entities. Guidelines were identified using the search terms fluoride varnish + [application; guidelines, or recommendations; children or pediatric; American Academy of Pediatrics (AAP); American Academy of Pediatric Dentistry] and a search of Guideline Central. Details of the guidelines were extracted and compared.

**Results:**

Ten guidelines met inclusion criteria. Guidelines differed in terms of periodicity recommendations and whether FV was indicated for children with a dental home or level of risk of dental caries.

**Conclusion:**

Numerous recommendations about FV delivery in medical settings are available to pediatric medical providers. Further study is warranted to determine whether the variation across current guidelines detected in this study may contribute to low FV application rates in medical settings.

## Introduction

Early childhood caries (ECC) is prevalent worldwide and is the most common chronic disease of childhood in the United States (US) (1). ECC risk is influenced by social determinants of health; Black, Mexican-American, Native American, and lower-income populations experience structural barriers to health in the U.S. and disproportionately higher rates of ECC (2). Children with ECC are at risk for substantial morbidity, including pain, infection, and school absences and parents of children with ECC suffer financial consequences from lost work days and costs for dental care (3). Fluoride varnish (FV) applications for children under the age of 6 years reduces ECC (4). The U.S. Preventive Services Task Force (USPSTF) first issued a recommendation (Grade B) in 2014 and again in December 2021 that primary care clinicians apply FV to the primary teeth of all children younger than 6 years of age beginning with the eruption of the first tooth, whether or not the child has oral health risk factors or a dental home (5, 6). The USPSTF is an independent volunteer panel of national experts in disease prevention and evidence-based medicine (7). The USPSTF conducts rigorous systematic reviews of the literature to inform their recommendations and is considered a leading independent source of guidelines for preventive health care. Despite good evidence for the effectiveness of FV in preventing ECC for all children under the age of six and minimal associated risk (6), <10% of eligible children are estimated to receive FV in pediatric primary care settings in the U.S (8).

Previously identified barriers to delivery of FV in medical settings include the time it takes to apply FV, lack of technical expertise, insufficient reimbursement, resistance from staff, and colleagues, and parental hesitance (9, 10). Small scale quality improvement interventions to improve delivery of fluoride varnish in medical settings have improved rates at the medical practice level but have not been widely disseminated (11–14). Although costs incurred by practices or patients could theoretically present an additional barrier, this service has been covered by most Medicaid, a publicly funded health insurance plan largely serving people with lower incomes, for more than a decade. Most commercial health insurers have covered FV application for children under 6 years of age since 2015 when the Affordable Care Act, which expanded health insurance coverage in the U.S., required all USPSTF recommended preventive services be covered without cost sharing by all insurers. Persistently low FV application rates and mixed results from an expansive quality improvement initiative (14) suggest that additional barriers to FV delivery in medical settings remain.

Clinical guidelines authored by different entities often exist for a given evidence-based practice (15). Variation in these the quality and content of these guidelines has been identified as a barrier to uptake of evidence-based interventions (16). The Institute of Medicine made recommendations for standards for development of trustworthy guidelines in 2008 (17), but guidelines' quality and consistency continues to be problematic (18). Differences in criteria for eligibility and periodicity for FV application in pediatric primary care settings has been detected at the level of state Medicaid programs (5), but whether variation in national FV application guidelines exists is not known. To address this gap in knowledge, we identified all current U.S. clinical guidelines for FV application for children under 6 years of age in medical settings, assessed consistency of the guidelines' content, and estimated the percentage of children for which FV application was indicated by each guideline (19).

## Methods

We identified currently available guidelines for FV application in medical settings for children under 6 years of age published by national pediatric or dental professional organizations or by national public health promotion entities that had been issued in the past 10 years. Guidelines were identified through a web-based search between February 1 and March 31, 2021, using the following search terms: fluoride varnish + [application; guidelines or recommendations; children or pediatric; American Academy of Pediatrics (AAP); American Academy of Pediatric Dentistry] and a search of Guideline Central. References for the guidelines identified through the web-based search were reviewed to identify any additional guidelines and the authors' prior research and clinical experience were used to assess the completeness of the list of guidelines. All publications that contained explicitly stated guidelines or made recommendations regarding FV application in medical settings by health care professionals for children under age six were included. If an organization had published multiple iterations of FV guidelines, only the most recent set of guidelines was included (e.g., we do not compare the 2014 guidelines released from the AAP Section on Oral Health because these guidelines were replaced in 2020). Guidelines were excluded if they only reported other guidelines and did not involve primary guideline development, were specific to other settings, such as dental practices or schools, had a focus other than a national population (e.g., state-level guidelines), focused on technical aspects of applying varnish, or had disclaimers.

The following data were extracted from each of the guidelines: (1) recommended periodicity of application; (2) whether periodicity recommendations depended on (2a) caries risk; (2b) dental home status, or (2c) consumption of fluoride in drinking water or as an oral supplement; (3) number of years the guideline has been in use; and (4) other guidelines referenced. We identified any guidelines referenced to understand whether guidelines were developed independently or relied in part on other guidelines to formulate recommendations. The extracted data were entered into a table and compared to the USPSTF guidelines and to each other to assess consistency across the guidelines.

To assess the potential impact of variation in guidelines, we estimated the percentage of children for which FV application was indicated by each guideline. These percentages were calculated by estimating the percentage of U.S. children <6 years of age with higher risk for ECC and the percentage with a dental home using national survey data (20, 21).

## Results

A total of 10 guidelines met criteria for inclusion (Table 1). Excluded guidelines were primarily those that only reported other guidelines, were intended for a non-national audience, or focused on technical aspects of application. The USPSTF guidelines are widely recognized in the U.S. as using rigorous methodology for guideline development and served as the comparator. Six of the 10 guidelines that met inclusion criteria recommended that FV application begin when the first tooth erupts and four did not specify when to begin application. The USPSTF guidelines stated that there was insufficient evidence to recommend a specific periodicity for application, but six of the 10 guidelines made three different periodicity recommendations. The USPSTF also stated that FV should be applied to the teeth of all children younger than 6 years of age, but six of the 10 guidelines indicated that, in addition to age, caries risk and/or presence of a dental home should be considered in determining whether or how often to apply FV. None of the guidelines recommended that exposure to fluoride in drinking water or oral fluoride supplementation should influence FV application decisions.

**Table 1:** Guidelines for fluoride varnish application in medical settings for children under six years of age.

**Organization issuing guideline and name of guideline**	**Periodicity**	**Specifies eligibility based on:**		**Guideline(s) referenced**	**Description of Guideline**
		**Dental home**	**Caries risk**		
**American Academy of Family Physicians**^**a**^ Website (22)	Endorses USPSTF recommendations			(1) USPSTF	Recommendations based on USPSTF systematic literature review.
**American Academy of Pediatrics**^**b**^**/Bright Futures** Periodicity schedule (23)	Every 3–6 months		X	(1) USPSTF (2) Fluoride Use in Caries Prevention in the Primary Care Setting	Succinct guide to timing of evidence-based pediatric preventive practices.
**American Academy of Pediatrics/Bright Futures** Infancy and early childhood health supervision visit guides 4th edition (24)	Every 6 months if no dental home^*^	X			Comprehensive guide for pediatric medical providers on evidence-based preventive/well-care.
**American Academy of Pediatrics/Bright Futures and National Inter-professional Initiative on Oral Health** Oral health risk assessment tool (25)	Not specified		X	(1) USPSTF	Tool that guides FV application in medical settings based on risk.
**American Academy of Pediatric Dentistry**^**c**^ Best practices: Fluoride Therapy (26)	At least every 6 months		X	(1) ADA Council on Scientific Affairs Expert Panel on Topical Fluoride Caries Preventive Agents	Recommendation is for “professionally applied” FV, not medical provider specific but cited in AAP Section on Oral Health Clinical Report advising on application of FV for children in medical settings.
**American Dental Association**^**d**^ **Council on Scientific Affairs Expert Panel on Topical Fluoride Caries Preventive Agents** Topical Fluoride for Caries Prevention (27)	At least every 3–6 months		X	(1) USPSTF (2) CDC - Other Fluoride Products	Cited in AAP section on oral health clinical report advising on application of FV for children in medical settings.
**Association of State and Territorial Dental Directors**^**e**^ Policy statement (28)	Not specified			(1) USPSTF (2) ADA Council on Scientific Affairs Expert Panel on Topical Fluoride Caries Preventive Agents	Summary of multiple guidelines and studies with general policy statement endorsing use of FV.
**Centers for Disease Prevention and Control**^**f**^ Other fluoride products, website (29)	At least twice per year				Guidance for use of fluoride products for caries prevention.
**Fluoride Use in Caries Prevention in the Primary Care Setting** AAP section on oral health clinical report (30)	Every 3–6 months	X	X	(1) USPSTF (2) AAPD best practices: fluoride therapy (3) ADA council on scientific affairs expert panel on topical fluoride caries preventive agents	Publication that provides guidance on evidence-based practices for pediatric health care providers.
**US preventive Services Task Force** Dental Caries in children from birth through age 5 years: screening (5)	Notes there is insufficient evidence to specify				Recommendations based on systematic literature review.

***(a) The American Academy of Family Physicians** is a non-profit medical society that represents 134, 6,000 family physicians, residents, and medical students*.

***(b) The American Academy of Pediatrics** is a non-profit medical society that represents 67,000 pediatricians and pediatric medical and surgical subspecialists. The Academy promotes optimal physical, mental, and social health for infants, children, adolescents, and young adults. It provides advocacy for children, health systems delivery research, public information and education, continuing medical education, and analyses and review of child health policy issues*.

***(c) The American Academy of Pediatric Dentistry** is a non-profit dental society that advocates policies, guidelines and programs that promote optimal oral health and oral health care for children*.

***(d) The American Dental Association** is a non-profit dental society with more than 162,000 members that aims to help dentists succeed and to support the advancement of the health of the public through advocacy and research*.

***(e) Association of State and Territorial Dental Directors** is a national non-profit organization representing the directors and staff of state/territorial public health agency programs for oral health*.

***(f) Centers for Disease Control and Prevention** is a federal government agency that is part of the Department of Health and Human Services. Its mission is to protect America from health, safety and security threats, both foreign and in the U.S. through education, research, public health programming and other initiatives*.

* *Dental home, presence of an ongoing relationship between a patient and dentist or dental practice*.

Inconsistencies were identified not only between different organizations' guidelines, but also within three distinct current sources of guidance on FV application published by the AAP. The *AAP/Bright Futures Periodicity Schedule* indicated that FV should be applied every 3–6 months and did not comment on dental home status, but the *AAP Health Supervision Guidelines, 4th Edition* screening tables for infants and young children and a *Clinical Report published by the AAP Section on Oral Health* in 2020 indicated that FV should only be considered if the child lacks a dental home.

**Figure 1:**
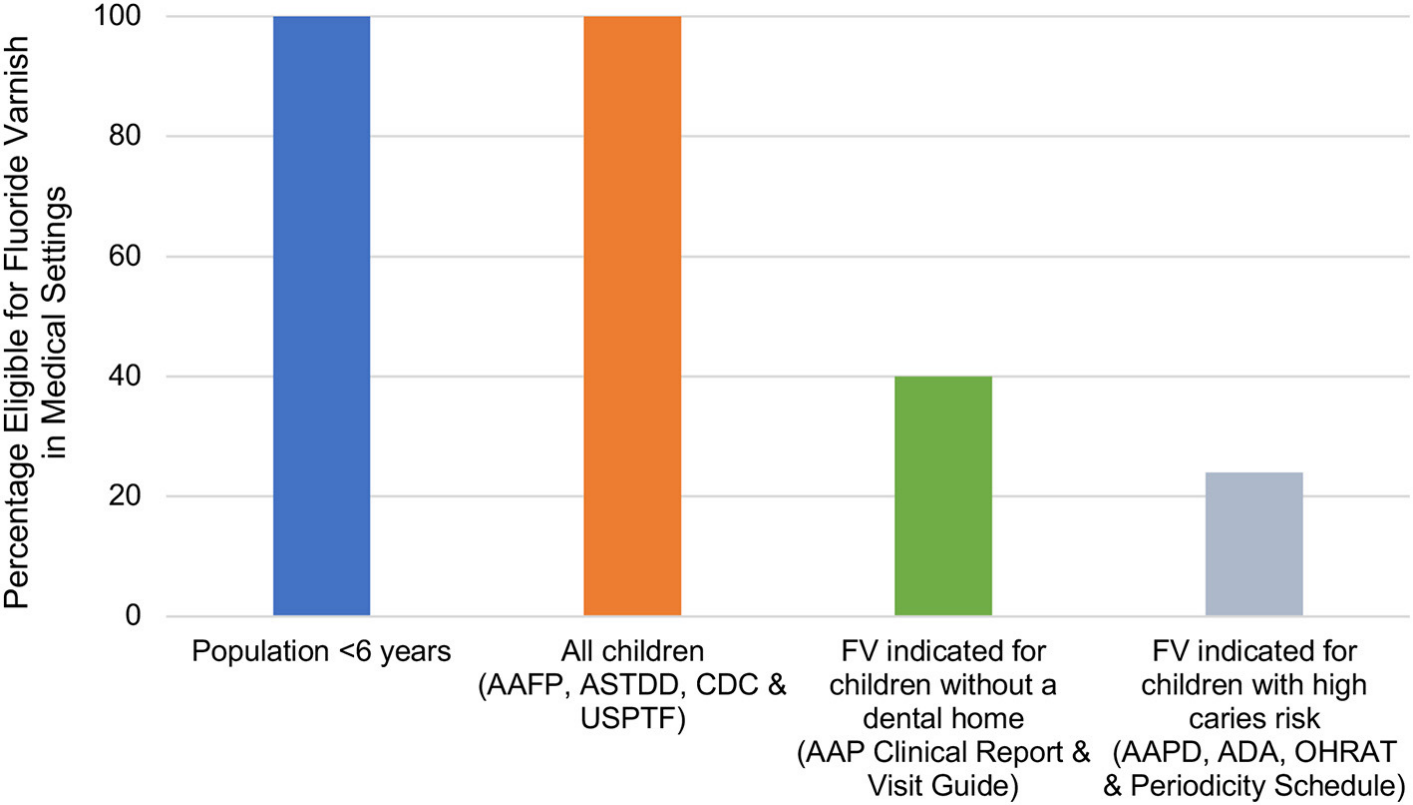
Estimated percentage of the population younger than 6 years of age eligible for fluoride varnish in medical settings by guideline. Estimates of children without a dental home were based on the 2016 MEPS, which found that about 60% of children aged 2–5 years had a dental visit in the past year (21). Estimates for children with high caries risk were based on the 2011–2014 NHANES, which found that about 24% of children aged 2–5 years had dental caries (20). AAFP, American Academy of Family Physicians; AAP American Academy of Pediatrics; AAPD, American Academy of Pediatric Dentistry; ADA, American Dental Association; ASTDD, Association of State and Territory Dental Directors; CDC, Centers for Disease Control and Prevention; OHRAT, Oral Health Risk Assessment Tool (AAP); USPSTF, U.S. Preventive Services Task Force.

Figure 1 illustrates the percentage of children for which FV application was indicated by each guideline, which was driven by guidance regarding caries risk or presence of a dental home. Of the 10 guidelines identified, four guidelines recommend FV for 100% of children under 6 years of age, two guidelines recommend FV for an estimated 40% of children under 6 years of age, and four guidelines indicate FV for an estimated 24% of children under 6 years of age.

## Discussion

Although FV application is recommended in medical settings for all children under 6 years of age based on evidence of benefit with minimal harm potential, coverage by all health insurers (5, 6), and cost-effectiveness (31–33), uptake of this intervention is low. This study identified variation in FV guideline recommendations in the U.S., a previously unidentified potential barrier to robust FV application in medical settings that serve young children. Guidelines differed on periodicity and whether or not providers should consider caries risk or presence of a dental home when determining if or how often to apply FV.

Pediatric health care providers are often parents' first source of information on oral health care for their children. In a 2014 survey of 402 pediatric medical providers, 41% felt they should provide FV to their patients' teeth, but only 7% did so for more than 75% of their eligible patients (34). In a study recently conducted by our team, we found that fewer than 4.8% of privately insured children under the age of six received FV during well-visits with their pediatrician (8), suggesting rates have not changed substantially. The ongoing low rates of FV application and high rates of ECC (8) represent an unsolved problem with implementation of an evidence-based oral health intervention for young children.

Health care providers rely on evidence-based, definitive, unbiased clinical guidelines to provide high quality patient care (18). An international systematic review of 216 pediatric clinical practice guidelines found that only 6.5% of the guidelines met the highest criteria for quality (“recommend”) based on the *Appraisal of Guidelines for Research and Evaluation II*, a systematic assessment of guideline quality (35). Prior studies of pediatricians' attitudes regarding and use of clinical guidelines showed relatively low use but were conducted more than 20 years ago (36, 37). When clinical guidelines vary as the current FV guidelines do, health care providers and parents must choose which, if any, of the guidelines to follow. Lack of consensus can generate uncertainty and may result in doubt regarding the intervention's benefit, resulting in low uptake, even if the intervention has a strong evidence-base.

The World Health Organization has identified dental caries as one of the leading non-communicable diseases in the world (38). Although replicating the current study for other countries across the globe was beyond the scope of the study and would be complicated by differing health care finance structures and regulatory environments, a brief exploration of guidelines for FV internationally suggest variation may be present there as well. A survey study by Bencze et al. found that the accessibility of clinical oral health prevention services such as FV application is limited and not free for children and varies across countries (39). A guideline on use of fluoride for prevention of ECC published by the European Archives of Pediatric Dentistry gives FV a “moderate” recommendation and recommends it primarily for children with higher risk for ECC (40) while Australian guidelines recommend FV for all children under 10 years of age (41).

The reasons for the low FV application rates in medical settings in the U.S. are likely multi-factorial and it will be important to continue to address previously identified barriers while further evaluating the potential role of guideline variation and identifying other potential barriers. Examples of multi-level interventions to improve FV application rates that could be tested include facilitation of referrals and inter-professional relationships between pediatric and dental practices and increasing the number of states that allow application by trained non-physician members of medical teams may increase FV application rates. State-level programs that provide training in how to apply FV, such as the Massachusetts Fluoride Varnish Training for Health Care Professionals Program (42), may also help to address other barriers. These interventions, along with guideline harmonization, may help to improve FV application in pediatric settings.

This study's strengths include its focus on clinical guidelines that did not require membership in a professional organization or institutional licenses to access, as these guidelines represent the those most broadly accessible pediatric medical providers. Other undetected barriers to FV application may contribute to low application rates. We were not able to assess pediatric medical providers' perceptions of the role of guideline variation in low application rates but this will be an important future study. This study also did not assess parents' acceptance of FV application in the medical setting, which may also play a role in low application rates. Finally, guidelines change frequently and new guidelines may have emerged since this study was conducted.

Increasing FV applications among young children is important because studies have shown that delivery of FV in medical settings can lead to improved oral health and cost-savings (43). Clear, comprehensive, high quality clinical guidelines facilitate uptake of evidence-based preventive services. Further studies are needed to determine how the variation among FV guidelines identified in this study may affect FV application practices in primary care pediatric settings in the U.S. Rapid evolution of scientific evidence can make it challenging to keep practice guidelines up-to-date, but periodic review and harmonization of FV application guidelines that take into account potential barriers to application may help to improve implementation of FV in medical settings for children under 6 years of age.

## Data Availability Statement

The original contributions presented in the study are included in the article/supplementary material, further inquiries can be directed to the corresponding authors.

## Author Contributions

SG and GG collected the data for the study. SG wrote the first draft of the manuscript. All authors contributed to revision of the manuscript and approval of its final form, contributed to the conception, and design of the study.

## Funding

This research was supported by the National Institute of Dental and Craniofacial Research (Grant No. R01 DE028530-01A1).

## Author Disclaimer

The content is solely the responsibility of the authors and does not necessarily represent the official views of the National Institute of Dental and Craniofacial Research or the National Institutes of Health.

## Conflict of Interest

GG and AK were employed by RAND Corporation, Arlington, VA, United States. AD was employed by RAND Corporation, Boston, MA, United States. The remaining authors declare that the research was conducted in the absence of any commercial or financial relationships that could be construed as a potential conflict of interest.

## Publisher's Note

All claims expressed in this article are solely those of the authors and do not necessarily represent those of their affiliated organizations, or those of the publisher, the editors and the reviewers. Any product that may be evaluated in this article, or claim that may be made by its manufacturer, is not guaranteed or endorsed by the publisher.
